# The impact of artificial intelligence on women’s empowerment, and work-life balance in Saudi educational institutions

**DOI:** 10.3389/fpsyg.2024.1432541

**Published:** 2024-09-30

**Authors:** Sayeda Meharunisa, Hawazen Almugren, Masahina Sarabdeen, Fatma Mabrouk, A. C. Muhammadu Kijas

**Affiliations:** ^1^Department of Management, College of Business Administration, Princess Nourah Bint Abdulrahman University, Riyadh, Saudi Arabia; ^2^Department of Economics, College of Business Administration, Princess Nourah Bint Abdulrahman University, Riyadh, Saudi Arabia; ^3^Wahaj Al-Itaqan Construction EST, Riyadh, Saudi Arabia

**Keywords:** artificial intelligence, AI infrastructure agility AI capabilities, work-life balance, women empowerment, structural equation modeling, organization efficiency

## Abstract

Gender prejudice and stereotypes are prevalent in the workplace, particularly for women in the Artificial Intelligence (AI) industry, where they can significantly hinder professional development and limit prospects for growth. These challenges contribute to the underrepresentation of executives in AI. However, with the right measures, these barriers can be overcome, leading to a more inclusive and diverse AI industry. Women in this demanding technological domain often face additional difficulties in achieving a work-life balance, further constraining their professional advancement and engagement in the industry. This research aims to examine the implications of AI capabilities on work-life balance and the empowerment of female faculty members in enhancing the efficiency of educational institutions. The research performs a structural equation modeling (SEM) approach, using a survey conducted on female faculty of Saudi Arabian universities. The study specifically considers moderating variables such as age, education level, experience, and marital status. The findings, which reveal that AI managerial capability, as well as AI infrastructure agility, impacts work-life balance and empowerment of women faculties in educational institution efficiency, underscore the significance of considering demographic factors when analyzing women’s empowerment and work-life balance as outcomes. By exploring these factors, the research provides a comprehensive understanding of how AI capabilities impact women’s empowerment and their ability to maintain a work-life balance, ultimately contributing to the efficiency and effectiveness of educational institutions. These results emphasize the value of increasing women’s empowerment and raising the standard of performance evaluation systems in educational sectors.

## 1 Introduction

A significant advancement in corporate management, artificial intelligence (AI) significantly impacts how workers operate, particularly in the human resources and employment departments (Choi, 2021; Havidz and Mahaputra, 2022; Kumar and Choudhury, 2022; Li et al., 2019; Murugesan et al., 2023; Pawlicka et al., 2020; Prentice et al., 2020; Shaikh et al., 2023; Wei and Li, 2022). Technologies based on AI have a different effect on how human resource management is done (Shukla and Bhasin, 2023). As an illustration, development can be created through a training program for every worker based on contextual procedures, real-time big data analytics, or data on employment practices. AI is the term that describes a technology that performs tasks that require some amount of intelligence to complete. It is a machine taught to perform tasks for people (Wang et al., 2022). When used effectively and practically, AI has improved the completion of human resource management work tasks, whether in hiring, performance evaluation, HR planning, employee training requirements, job evaluation, or even forecasting the labor market’s needs and indicators (Ahmad and Mustafa, 2022). According to a review by the top provider of cloud-based applications for sector-specific applications, with the speed at which technology is changing, already starting to see examples of innovative use of AI in ways that might enhance workflow are evident (Budhwar et al., 2022). Regarding the job done by those employed in the recruitment and human resources divisions (Sharma et al., 2022), several businesses and organizations have already proven AI’s potential to raise care quality and/or lower costs.

One of the most pressing issues of the twenty-first century is women’s empowerment (WE), which is improving women’s economic, social, political, and cultural standing, especially those underprivileged in society (Infante and Darmawan, 2022). Women have the power to transform and influence their families and, by extension, the economy. Women have, however, long faced numerous obstacles in terms of culture, economy, education, and the workplace. It is important to know that social assistance laws mandate that women lose their authority if they are unable to access necessary resources, earn an income, or save money (Kumari and Eguruze, 2022). The protection and upholding of women’s rights in the areas of welfare, education, and workforce has led to a global trend in women’s empowerment. In addition, women’s empowerment fosters gender equality, enabling them to participate equally in the processes of economic growth and development (Youssef, 2022). For the world to be prosperous and sustainable, gender equality and empowerment are crucial, based on the UN’s 17 Sustainable Development Goals (SDG). One of the projects seen to be able to provide women engineers more control over their lives and professions is the empowerment of women engineers in the energy sector (Cakranegara et al., 2022). Having total respect, being independent, and living a happy life are all signs of empowerment. Therefore, empowering women will help them to become engineers and realize their full potential (Lee et al., 2023). The work-life balance for employment and female empowerment are significantly correlated. Their ability to strike a work-life balance determines their decision to work and their level of career achievement (Kendeikyo, 2022).

In today’s competitive world, organizations face serious hurdles in surviving (Abendroth and Den Dulk, 2011). The biggest issue among these is keeping talented employees (Haar et al., 2014). Business organizations are creating policies regarding hiring, selection, onboarding, training and development, pay and benefits, job design, evaluation of the job and wage standards, etc. to combat the attrition problem. These policies ultimately aid the organizations in keeping their workforces (Sham et al., 2021). According to a research study, HRM practices like effective leadership, communication, and value profiles need to be integrated with strategic goals that help improve women’s empowerment and the work-life balance of working women (Rahaman and Tul-Jannat, 2015). Employee well-being refers to how an employee is looked after by the company. It’s a feeling of security within the company. AI and other technological breakthroughs have made it easier to retain talent well-being.

AI can speed up the educational process and help students accomplish their goals. It gives students access to the appropriate course, enhances their interaction with faculty, and enables them to focus on other facets of their lives by saving them time. Personalization, a crucial feature of AI, enables students to take a personalized approach to learning based on their particular skills, preferences, and life experiences (Chen et al., 2020). To ensure that students get the most out of their education, AI adjusts itself to their level of knowledge, pace of learning, and preferred goals. In addition, AI can assess students’ prior academic performance, pinpoint their areas of weakness, and enhance upcoming learning opportunities for a tailored learning experience. It also gives faculties more control by automating chores like paper grading, administrative work, and learning pattern evaluation (Mabrouk et al., 2023; Mabrouk et al., 2022).

Therefore, the primary goal of this research is to identify the importance of AI and its effects on work-life balance and empowerment of female faculties in educational institution efficiency in Saudi Arabia. To achieve this objective, we formulated the following research questions: Does artificial intelligence significantly impact the balance of work-life and empowerment of female faculties in educational institutions? And do the balance of work-life and empowerment of female professionals have any relationship?

By examining these interconnected elements of AI, women’s empowerment, and employee work-life and well-being this research aims to provide a comprehensive understanding of how AI capabilities impact the empowerment of female faculty members and their ability to maintain a work-life balance, ultimately enhancing, their well-being and the efficiency of educational institutions. Gender prejudice and stereotypes are prevalent in the workplace, particularly for women in the AI industry, where they can significantly hinder professional development and limit prospects for growth. These challenges contribute to the underrepresentation of female executives in AI. However, proper measures can overcome these barriers, leading to a more inclusive and diverse AI industry. Women in this demanding technological domain often face additional difficulties in achieving a work-life balance, further constraining their professional advancement and engagement in the industry. The study specifically considers moderating variables such as age, education level, experience, and marital status. By exploring these moderating factors, the research offers a thorough grasp of how AI capabilities affect women’s empowerment and work-life balance, thereby enhancing the efficacy and efficiency of educational establishments.

Several additional studies have suggested that AI investigates work-life balance and that women’s possibilities inside the organization should be expanded upon. Moreover, the common studies examining the connections between AI and employee outcomes have been carried out in the context of a global company. Furthermore, the degree to which intelligent technologies influence the industry’s adoption of digital human resources can also be influenced by national culture.

Moreover, while studies on women’s empowerment and work-life balance exist, they tend to treat these issues in isolation, without adequately considering the moderating role of demographic factors such as age, education level, experience, and marital status. This oversight is particularly evident in the AI industry, where the intersection of these variables with AI capabilities has not been thoroughly explored.

Furthermore, there is a lack of research that connects these elements to the efficiency and effectiveness of educational institutions, despite the growing influence of AI in shaping modern educational practices. This study seeks to fill these gaps by exploring the complex interplay between AI capabilities, women’s empowerment, work-life balance, and the efficiency of educational institutions, with a focus on the moderating effects of key demographic factors.

A literature review is done to achieve these goals. The research hypotheses are formulated and tested empirically on a sample of women faculties in Saudi Arabia Universities. The last section contains the conclusions drawn from the investigation. Thus, in addition to the literature, the study will benefit Saudi Arabian universities. A conceptual framework will also be suggested for further research in this paper.

The remaining portion of the work consists of literature reviews in the second section, which reviews the existing works to fulfil the investigation goal. The third section explains the research methodology, the fourth section presents the findings and their interpretation, and the last and fifth part explains the conclusion and policy recommendations.

## 2 Literature review

AI deals with developing and applying computer systems that can solve problems that often call for human intelligence (Haton, 2006). AI techniques were recognized as relevant to computer-assisted learning. Drill-and-practice computer programs were prevalent in early educational computer applications. Systems facilitate user-to-user communication in natural language, as well as those that are generative, adaptive, or self-improving (Stubbs and Piddock, 1985). AI systems mostly work with symbolic data to solve tasks, rather than just numerical data. AI heavily utilizes a variety of types of domain-specific knowledge, such as natural language processing, speech recognition and understanding, image interpretation and vision, and robotics. AI is applied in a wide range of industries, including banking & finance, industry, and medicine (Haton, 2006).

AI is a new technology that has taken the shape of natural learning processing, machine learning, human-machine interaction, and other things. It was first widely used in the corporate world and, more recently in higher education. Higher education institutions’ primary goal is to test new technological innovations and profit from the resulting system. But it’s only marginal because AI has a long way to go before teaching and learning processes can be automated or replaced by robots in place of instructors (Ayed, 2022).

The goal of the broad field of AI is to automate tasks that now need human intelligence. Computerized medical diagnosticians and systems that automatically adapt hardware to specific user requirements are examples of recent advances in AI. The five main issues that AI addresses are perception, manipulation, reasoning, communication, and learning. Creating representations of the real world from sensory data is the focus of perception (Williams, 1983).

The fundamental idea of AI technology is the idea that human intelligence is researched and replicated, allowing the programming of computers to carry out employment possibilities that individuals perform. This study reviews the literature within the framework of AI applications in management and human resources to fulfil the goals of this investigation.

### 2.1 Review of artificial intelligence and work performance

Recent research emphasized the potential of AI and work performance in various sectors. Li et al. (2019) examined the impact of a hotel employee’s intention to leave on their awareness of robotics and AI. Additionally, they illustrated the moderating effects of the emotional atmosphere of competition and supposed structural funding. Utilizing information obtained from 468 samples of full-time staff members at a 5-star hotel in Guangzhou, China. It was discovered that employee turnover intention was highly connected with an awareness of AI besides robotics. The outcome demonstrates that the link was constrained by the psychological climate of competition and perceived organizational support. Similarly, Prentice et al. (2020) examined the impact of emotion and AI on worker productivity and retention with an emphasis on hotel service workers. They also evaluated that employee performance translates into both internal and external operational forms aspects that quantify how people during interactions with both internal and exterior service providers fulfill the objectives of customers and coworkers, accordingly. The information was gathered from numerous rated hotels. The outcome indicated that demonstrative intelligence has a large impact on employee retention and performance, whereas AI has a substantial influence on employee presentation. Moreover, Choi (2021) exposed the function of AI at a front-line service meeting to learn how users react to services powered by AI. The researcher used online surveying to gather information from 454 Korean employees, and then hierarchical regression to test the theory analytically. Initially, the outcomes showed that people are more inclined to accept AI technology when the responsibilities of the user and the AI are clear. The findings demonstrated that role clarity and user acceptance of technology used in AI are weakened by the deployment of AI-based technologies. Additionally, capability and consumer readiness, the trust engendered by the deployment of technology based on AI, make it easier for people to embrace it.

In addition, Pawlicka et al. (2020) explored the variables influencing a person’s subjective perception of their work-life balance with a machine learning technology among 80 randomly chosen organizations. This was accomplished using the information provided by a huge group of employees in Poland. They found a correlation between the feeling of balance and the actual working hours with work-life balance. They observed that the perception of equilibrium and the definite employed times were the most important factors; changing, it caused the tool to forecast the transition from balance to imbalance. The outcomes might be used to forecast and stop the foreseeable occurrence of a conflict between work and life.

Furthermore, a study Kumar and Choudhury (2022) explored the relationship between women, the application of technology, and potential futures in developing countries. The information was gathered from 125 female volunteers and 100 male volunteers, mostly from South Asia, who worked in poor countries. The results demonstrated a substantial and favorable intra-correlation between variables, including attitudes toward AI robots, requirements for using robot gender, AI-based machines, and the threat posed by AI robots.

Wei and Li (2022) investigated the effects of artificial intelligence on manufacturing workers’ mental health. Additionally, they illustrated the part of mediation between actively working and the workplace. They examined how AI affected factory workers’ depression symptoms using information from the China Labor Force Dynamics Survey in 2018, and they tested whether the presence of overtime work has a mediation influence on the workplace environment using stepwise and bootstrapping methodologies. The findings indicated that AI benefits workers’ mental health since it can lower the psychological depression ratings of manufacturing workers.

Another study by Murugesan et al. (2023) outlined the benefits of AI to industry practices and HR digitization. The analysis focuses on 5 AI applications in HR competence and 3 components of HR preparedness, including 271 manufacturing, IT, and administrative HR experts. The SPSS tool and the AMOS program were used for the data acquired. The results indicated that one of the most important steps in establishing sustainable growth is to examine hierarchical organization.

Similarly, Shaikh et al. (2023) examined the effect of AI on worker productivity. They also assessed how well-being and information exchange mediated the relationship. In Pakistan’s largest hospitals, 184 doctors provided the data. Results from partial least squares analysis suggest a causal link between AI and worker productivity. The findings confirmed that AI and information exchange, mental health, and well-being underpin employees’ productivity.

### 2.2 Review of artificial intelligence in educational institutions

Since there are many types of labor demands in the higher education sector, including research objectives, student performance and satisfaction reports, and administrative aims, this sector is suitable for the research. Comparably, to satisfy the demands of quality work, a variety of skill sets are needed. Role conflict between work and family is inevitable, as quantitative job demands rise (Bakker and Demerouti, 2018; Naidoo-Chetty and du Plessis, 2021; Theron et al., 2014). Work–family conflicts arise from these inter-role conflicts and the blending of personal and professional spheres, which have a detrimental effect on female administrators and their employers (Havidz and Mahaputra, 2022; Shukla and Bhasin, 2023). In particular, Arabic society perpetuates the assumption that women belong at home and should take care of the family. As a result, women in administrative roles face pressure to balance work and family responsibilities. Women are advised to choose their place of employment, but they are still expected to prioritize taking care of their families and meeting social obligations (Turanlıgil and Farooq, 2019).

A set of studies on AI and its impact on educational institutions were carried out in Arab regions. Melweth et al. (2024) evaluated burnout, technological confidence, and perspectives on AI integration among secondary school teachers in Asir, Saudi Arabia. They found that experience and gender were highly correlated with burnout and technology confidence. Qualitative results revealed circumstances that lead to teacher stress. This study highlights the importance of teacher-centered integration policies that start with minimal automation of administrative tasks, engage teachers in the digital transition as partners, prioritize welfare over test scores, and utilize localized research to guide solutions. Moreover, another study in Jordan stressed that society and women in technology stand to gain from supporting and empowering women in academics, particularly at the entry level. These factors make it necessary for Jordanian colleges to make plans to assist women who work in academia and in the technology sector particularly (Ata et al., 2022).

In addition, Alotaibi and Alshehri (2023) outlined that the integration of AI has become a critical goal for many higher education institutions in Saudi Arabia as part of the 2030 Vision for Comprehensive Development of Higher Education. The purpose of this study was to investigate the advantages and disadvantages that Saudi Arabia’s higher education institutions face when they implement AI-based learning outcomes. The findings emphasized that AI is still in its infancy as it relates to learning, but it is now an indisputable fact for universities. Accepting and utilizing this revolutionary technology will be essential to addressing upcoming learning obstacles, and every student must have the technical know-how required to communicate with and develop artificial intelligence in the future. Another study (Muafa and Al-Obadi, 2024) investigated the advantages and disadvantages that Saudi Arabia’s higher education institutions face when they implement AI-based learning outcomes. The findings emphasized that AI is still in its early stages as it relates to learning, but it is now an indisputable fact for universities. Similarly, Al-Abdullatif et al. (2023) urged more research to determine how well chatbot supports teaching and learning processes, a substantial contribution to the research on incorporating it in institutions of higher learning.

Furthermore, Alkinani (2021) assessed the assimilation and implementation of digital technology in education expands the prospects for Saudi university students and instructors to navigate effectively the increasingly interconnected digital world. However, the study identified the three categories of challenges. (1) Barriers at the teacher level (attitudes, knowledge, access, and resistance to change). (2) Institutional hurdles, perceived utility, compatibility, and (3) barriers at the technology level and institutional level, such as resources and support from leaders. Therefore, the researcher proposed that AI is expected to become the core of higher education in Saudi Arabia.

Comparatively, some research has been carried out in the UAE, among other Arab regions. A study of work-life imbalances and resulting outcomes is presented from the perspective of both employees and organizations in the UAE. To present a complete view of work-life imbalances in higher education, it combines spillover and facilitation theories. A key aspect of the study is how work-life imbalances are affected by social demands when they outweigh work demands in socially demanding societies. It illustrates the tension between work-life imbalances and personal career goals (Begum et al., 2024). In the meantime, Heath (2021) emphasized that incorporating women into AI is crucial to its effective development, use, and governance in the UAE. Moreover, a study by Mahmood et al. (2023) investigated the leadership experiences of a diverse group of women in the United Arab Emirates, looking at the variables that affect their ability to lead successfully. Considering the distinct organizational, technical, and cultural aspects of the United Arab Emirates. The results demonstrate the beneficial effects of AI training and access to cutting-edge technology resources on leadership achievement. They also underscore the significance of an encouraging corporate culture and the growing need for technological competence.

A recent similar study by Iyer et al. (2024) examined the potential benefits of new technologies by evaluating big data on student learning patterns, engagement levels, and performance. The findings might lead to the development of tailored learning pathways, adaptive courseware, and focused interventions. Virtual laboratories and AI-powered tutoring programs will soon provide individualized and immersive learning experiences. Campus operations may become more sustainable and effective by using IoT sensors to monitor and control resource allocation, building security, and energy use. Systems with AI capabilities may automate administrative work, optimize workflows, and offer facilities with predictive maintenance. In this study, they focused on individual learning factors, system thinking factors, and connective factors at Dubai University in general. However, there is scant literature focusing on AI and empowerment and work balance in educational institutions in the Arab region in general, Saudi Arabia specifically. Our study will fill this gap in the literature.

### 2.3 Conceptual background and research hypothesis

**Figure 1:**
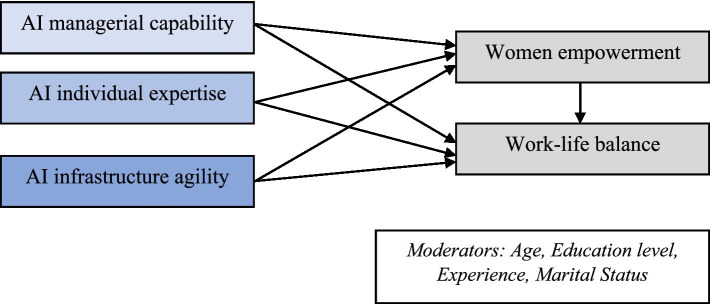
Research framework. Source: Authors’ presentation.

Even though there is still very little released study on the economic cost as well as application of AI in the structural situation, various studies have found barriers to the success of implementations in artificial intelligence initiatives. The goal of the study is to observe the artificial intelligence capabilities in work-life balance and empowerment of female faculties (Figure 1). A framework for the research hypothesis based on earlier publications (Wamba-Taguimdje et al., 2020b) was used to analyze the data.

AI capabilities could be defined as the firm’s ability to create a bundle of organizational, personnel, and AI resources for business value creation and capture (Anand and Fosso Wamba, 2013; Kim et al., 2011). Various IT categories have already been utilized in earlier studies. An organization can use organizational, material, immaterial resources, and human. In this analysis, we consider three categories of AI resources based on prior research.

The capacity of an association and the situation in which employees control or simulate behavioural intelligence in an electronic device or other technological device to generate price for the sustainability of the company is known as AI Management Capability (AIMC). AI management capability potential is peculiar to strategic planning, strengthening relationships within and between companies, investment decision-making, coordination, and control (Dubey et al., 2021; Giraud et al., 2023). The managerial capacity of AI has a potential that is unique to “strategic planning, strengthening relationships within and between organizations, investment decision-making, coordination, and control” (Wamba-Taguimdje et al., 2020b). Kim et al. (2011) have demonstrated in their study that control, which depends on the expertise of the staff, has influences on infrastructure flexibility.

*H1*: AI managerial capability significantly influences the women’s empowerment and work-life balance of women’s faculties in educational institutions.

AI personal Expertise (AIPE), is defined as the professional skills and knowledge of AI-related technologies, business functions, and relational (or interpersonal) domains required by the organization’s staff for modeling and/or using intelligent behavior in a computer or other technology to accomplish the tasks assigned to it (Giraud et al., 2023). To manage the AI properties at their clearance more effectively, an administration of IT professionals must possess a range of abilities, including consciousness, possession, incorporation, organization, and knowledge of IT components. As a result, the ability of AI with an organization’s strategy is crucial for the development of commercial value; staff members who possess the correct mix of talents enhance this efficacy. However, the expertise of AI staff becomes an intangible asset for organizations when IT staff understands how the organization’s strategies are mixed with IT and AI skills (Dubey et al., 2021). Institutions with knowledgeable AI experts are, therefore, more likely to produce trustworthy and affordable intelligent systems that will fulfil the demands of dynamic, ever-changing surroundings. Consequently, we can state the following suggestion:

*H2*: AI individual expertise significantly influences the women’s empowerment and work-life balance of women’s faculties in educational institutions.

AI infrastructure Flexibility (AIIF), refers to the composition of all technological assets (software, hardware, and data, etc.), systems and their components, network, and telecommunications installations, and applications that are necessary for the implementation of an AI system capable of performing tasks (Markolf et al., 2021). The adaptability of installing AI infrastructure for organizational operations makes the ability to quickly support different scheme mechanisms and adjust to altering situations and approaches, such as financial constraints, planned associations, mergers and acquisitions international collaborations, or mergers possible. Multiple key elements must be brought together to ensure the success of AI in an organization (Wamba-Taguimdje et al., 2020a). Information-coupled IT and AI talent, field expertise and technology, important choices, outside alliances, and ascendable structure are some of them. The first four components are the engine’s fuel and adaptable infrastructure, deprived of which anything can function. A better IT infrastructure allows institutions to use IT resources effectively and efficiently, to support structural restructuring through the deployment of AI technologies (Wamba-Taguimdje et al., 2020a). A self-adjusting, repairing, and improving network will support proactive performance improvement and resource optimization, encourage strategic company procedure innovation, and prevent issues before they arise. Consequently, we can state the following suggestion:

*H3*: AI infrastructure agility significantly influences the women’s empowerment and work-life balance of women’s faculties in educational institutions.

Empowerment enhances women’s ability to decide and choose what is best for them to reduce their double burden (Syahirah Syed, 2010). The equal opportunity and equal participation of women relative to men increases the ability of women to have a greater work-life balance (Syahirah et al., 2015). This is because women have control in managing their work and personal lives, since their voices and demands are counted by the employer. As mentioned in Andreassi and Thompson (2008), equal opportunity urges the organization to improve the status of women employees through the implementation of organizational support for work-life balance. According to Akdağ (2012), empowerment is one of the key factors of work-life balance. The sense of gaining control positively affects the mental, emotional, and physical of individual, thus directly resulting in the balance between work and personal life (Akdağ, 2012).

Empowerment enhances women’s ability to decide and choose what is best for them to reduce their double burden (Syahirah Syed, 2010). The equal opportunity and equal participation of women relative to men increases the ability of women to have a greater work-life balance (Syahirah et al., 2015). This is because women have control in managing their work and personal lives since their voices and demands are being counted by the employer. As mentioned in Andreassi and Thompson (2008), equal opportunity urges the organization to improve the status of women employees through the implementation of organizational support for work-life balance. According to Akdağ (2012) empowerment is one of the key factors of work-life balance. The sense of gaining control positively affects the mental, emotional, and physical of an individual, thus directly resulting in the balance between work and personal life (Akdağ, 2012).

Empowerment enhances women’s ability to decide and choose what is best for them to reduce their double burden (Lekchiri and Eversole, 2021). The equal opportunity and equal participation of women relative to men increases the ability of women to have a greater work-life balance (Dajani and Moustafa, 2021). This is so that women may manage their own private and work lives since their voices and stresses are occupied into explanation by the company. Equal opportunities request that the company improve the status of female employees by implementing organizational encouragement of balancing work and life. According to Uddin (2021), empowerment is one of the key factors of work-life balance. The feeling of taking back control has a favorable effect on one’s emotional, mental, and physical well-being, which directly contributes to the harmony between work and personal life.

*H4*: There is a significant relationship between women’s empowerment and the work-life balance of women’s faculties in educational institution settings.

The relationships between AI managerial capability, AI individual expertise, and AI infrastructure agility with women’s empowerment and work-life balance are likely impacted by various demographic factors such as education level, age, marital status, and experience. Age, as a moderating variable, may influence these relationships by altering how employees respond and perceive women’s empowerment and work-life balance (Chakraborty et al., 2020; Mukanzi and Senaji, 2017; Sun et al., 2023). Senior employees may value different aspects of women’s empowerment and work-life balance compared to younger employees, thereby weakening or strengthening these relationships. Correspondingly, education qualification can shape the thoughtful utilization of managerial decision-making and characteristics, individual determination, and ability, hypothetically moderating their impact on women’s empowerment and work-life balance. Highly educated employees might leverage these aspects more effectively, leading to significant relationships (Aruldoss et al., 2022; Sun et al., 2023). Work experience, as another moderator, is anticipated to impact these relationships by leveraging employees with a wide range of perspectives and a profound understanding of workplace dynamics, possibly enhancing the influence of AI managerial capability, AI individual expertise, and AI infrastructure agility with women’s empowerment on work-life balance (Chakraborty et al., 2020; Mukanzi and Senaji, 2017). Lastly, the marital status of the employees may also moderate these associations, as marital assurances and responsibilities can impact how employees engage with their tasks in the workplace and experience happiness or well-being. Thus, it is hypothesized that age (H5), education level (H6), experience (H7), and marital status (H8) will moderate the relationships from H1 to H4, leading to varying levels of influence on women’s empowerment and work-life balance (Alsulami et al., 2022; Aruldoss et al., 2022; Chakraborty et al., 2020; Mukanzi and Senaji, 2017; Sun et al., 2023).

*H5*: Age moderates all relationships from H1 to H4.

*H6*: Education level moderates all relationships from H1 to H4.

*H7*: Experience moderates all relationships from H1 to H4.

*H8*: Marital status moderates all relationships from H1 to H4.

### 2.4 Proposed research framework

The structure listed below is suggested for investigation in subsequent studies based on earlier investigations and a literature review. The framework is grounded in the empowerment theory by Zimmerman (2000). The empowerment theory provides methods for analyzing the concept in various contexts and for researching the empowering procedure. For instance, this model enables the investigator to examine the various organizational, personal, and social contexts of empowerment. Empowerment allows individuals and organizations to see a closer relationship between their goals and how to achieve them (Mechanic, 1991). It demonstrates the link between life and work outcomes that influence a person’s life. Applying this theory could require changes to organizational structure and processes, which would increase employee access and participation and boost the efficiency of the organization in achieving goals. When applied to the study, this theory shows how the welfare, access, conscientization, involvement, and control of women affect their ability to trust work also family obligations. Balancing work and life is regarded as a factor influencing a personality’s quality of life. In reality, also has an impact on whether or not female facilities decide to stay in the field. Figure 1 shows the framework of the proposed conceptual model.

## 3 Research methodology

The investigation of objectives and the testing of hypotheses depend on the accuracy with which the variables are measured, and then on the techniques and procedures used to conclude.

### 3.1 Sample design

The female faculties of Saudi Arabian universities are selected as the research sample. The researcher selected women employees working in educational sectors are the sample frame.

### 3.2 Sampling method, sampling size

The population of this study consists of female faculty members from various universities across Saudi Arabia. The decision to focus on female faculty members was made due to (1) Gender-Specific Challenges: Female faculty members often face unique challenges in their academic and professional environments, including gender bias, work-life balance issues, and cultural expectations that may differ from those of their male counterparts. These factors can significantly impact their physical and mental well-being, making it important to understand their specific experiences (Groenewald et al., 2024; O’Connor and Liu, 2024). (2) Underrepresentation in Research: There is a lack of research focusing on female academics in Arab countries, especially Saudi Arabia, regarding their well-being when securing their position between family and work. By focusing on this group, the study aims to fill this gap and contribute to the broader understanding of gender dynamics in academia (Ballantyne, 2022; Herbst, 2020; Wilson et al., 2020). (3) Cultural and Social Context: (Al-Qahtani et al., 2020; Mabrouk et al., 2022) Studying this group allows exploring how these norms influence their professional lives and well-being.

The sample was selected using a random sampling method to ensure that each female faculty member within the selected universities had an equal chance of being included in the study. This approach enhances the representativeness of the sample and reduces selection bias. To calculate the required sample size, the study considers a confidence level of 95%, and a margin of error of 8% to ensure a representative group within this population. The latest open data from the Saudi Ministry of Education shows that female faculties in Saudi Universities represent 40.8% of the total population and equal to 34,841. Based on this data, the required sample is 145. The sample size of this study consists of 180 participants and provides valuable insights into the experiences and perspectives of this specific group.

### 3.3 Data collection

For the study’s purpose, primary data are gathered. Primary data for the study is gathered using the survey approach. With the aid of a specially created questionnaire and in-person interviews, the necessary information is gathered from the sample respondents.

### 3.4 Questionnaire design

A questionnaire is created based on the study’s goals. Using a standardized questionnaire, the primary data is gathered. To learn the thoughts of female faculty members, a questionnaire including demographic information and a Likert five-point scale was employed, along with some closed- and open-ended questions to gather appropriate responses.

### 3.5 Analysis tool

Based on statistical analysis utilizing the AMOS and SPSS, an analysis was carried out. The measurement and structural models are assessed simultaneously by structural equation modeling, leading to concurrent factor analysis and hypothesis testing.

## 4 Analysis and interpretations

### 4.1 Demographic profile

We circulated a Google survey among the universities and received 180 completed questions. In that 76.7 (*n* = 138) percent of faculties were married and the remaining 23.3 (*n* = 42) percent of female faculties were unmarried. Also, a maximum of 41.7 (*n* = 75) per cent of women faculties were between the ages of 41 to 50 years, 36.1 (*n* = 65) per cent of women faculties were between the ages of 31 to 40 years, 16.7 (*n* = 30) per cent of female faculties were in the age of 21 to 30 years, and remaining 5.6 (*n* = 10) per cent of female faculties were above the age of 50 years. Furthermore, a maximum of 57.8 (*n* = 104) per cent of female faculties were professionals and also, 46.1 (*n* = 83) per cent of faculties had less than one year of experience in their sector, 26.1 (*n* = 47) per cent of faculties had 1 to 5 years of experience, 22.2 (*n* = 40) % of faculties have 5 to 10 years of experience, and 5.6 (*n* = 10) per cent of faculties have 10 years and above work involvement. The demographic profile of female faculties is revealed in Table 1.

**Table 1:** Demographic profile.

Variables	Constructs	Per cent	Frequency (*n*)
Marital status	Married	138	76.7
	Unmarried	42	23.3
Age	21 to 30 years	30	16.7
	31 to 40 years	65	36.1
	41 to 50 years	75	41.7
	Above 50 years	10	5.6
Education level	UG	6	3.3
	PG	70	38.9
	Professionals	104	57.8
Work experience	Less than 1 year	83	46.1
	1 to 5 years	47	26.1
	5 to 10 years	40	22.2
	Above 10 years	10	5.6

Source: Authors’ calculation.

There were 192 questionnaires in total, and 180 of them were utilized. The data were loaded into the SPSS program to be ready for hypothesis testing by conducting scale validity and reliability analysis. Structural equation modeling (SEM) was used for the data analysis in this study, using the two-step approach recommended by Anderson and Gerbing (1988). We tested the measurement models first to make sure the observed variables were adequately connected to the appropriate variables for each scale. Reliability, validity, and confirmatory factor (*CF*) analyses were verified in the measurement approach to find the consistency, validity, and final measurement model for each latent construct. Second, the proposed structural model was examined using SEM. The AMOS v23 software was used for the analyses and maximum likelihood estimation. Then, the hypothesis is tested by performing regression analysis.

**Table 2:** Reliability of measures.

Variables	Cronbach’s Alpha
Women Empowerment (WE)	0.808
Work-Life Balance (WB)	0.825
AI Managerial Capability (MC)	0.889
AI Individual Expertise (IE)	0.884
AI Infrastructure Agility (IA)	0.823

Source: Authors’ calculation.

### 4.2 Measurement model

Initially, the reliability of the construct is tested and demonstrated in Table 2. The evaluation of the level of consistency between different variable measures is related to authenticated analysis. The dependability of the variables across all dimensions is assessed using Cronbach’s Alpha coefficient. Bagozzi and Yi (1988) are among those who suggested the values of all variable or dimensional scales to be greater than the commended value of 0.60. The reliability of the measured compound is shown by the fact in this study, Cronbach’s alpha coefficients were > 0.60 for all the examined factors. Table 3 presents the measures’ alpha value. In that, for each construct, the value of Cronbach alpha is greater than 0.60, i.e., Women Empowerment (0.808), Worl-Life Balance (0.825), AI Managerial Capability (0.889), AI Individual Expertise (0.884), and AI Infrastructure Agility (0.823). Hence, it is concluded that all the measures are reliable and consistent.

**Table 3:** Measures with composite reliability, factor loadings, and AVE.

Variables	Constructs	FL	CR	AVE
Women Empowerment (WE)	WE1	0.677	0.756	0.6101
	WE2	0.732		
	WE3	0.772		
	WE4	0.827		
	WE5	0.621		
Worl-Life Balance (WB)	WB1	0.760	0.907	0.5140
	WB2	0.640		
	WB3	0.733		
	WB4	0.751		
	WB5	0.767		
AI Managerial Capability (MC)	MC1	0.809	0.886	0.6974
	MC2	0.778		
	MC3	0.677		
	MC4	0.610		
	MC5	0.535		
AI Individual Expertise (IE)	IE1	0.637	0.933	0.6570
	IE2	0.768		
	IE3	0.809		
	IE4	0.641		
	IE5	0.687		
AI Infrastructure Agility (IA)	IA1	0.500	0.732	0.5852
	IA2	0.610		
	IA3	0.729		
	IA4	0.837		
	IA5	0.834		

Source: Authors’ calculation.

Also, the measurements’ dependability is tested using Composite Reliability (CR), Factor Loadings, and Average Variance Extraction (AVE). The factor loadings (FL) that were > 0.7 were kept, while the others were eliminated. Only items that satisfy the factor loading requirement are included in Table 4. To assess the scale’s applicability to our data sample, it is helpful to know the average variance extracted or AVE and composite reliability or CR values. Our values satisfied the threshold for an acceptable value of AVE, which is defined in the literature as 0.5 or higher. For each variable, the value of CR should be above 0.7, and the value of CR for our variables indicated by the results also meets the requirement (Singh, 2017).

**Table 4:** Hetero-trait Mono-trait (HTMT) ratio of measures.

Variables	WE	WB	MC	IE	IA
WE	0	0	0	0	0
WB	0.5699	0	0	0	0
MC	0.3493	0.9509	0	0	0
IE	0.4185	0.5279	0.4984	0	0
IA	0.6228	0.7036	0.6414	0.7254	0

Source: Authors’ calculation.

From the Table 4, it was found that the FL for all the factors is >0.50, the CR of all the constructs was >0.7, and the average extraction of variance value for all the factors was >0.5. Hence, concluded that all the measures are highly dependable for performing further analysis.

The Hetero-trait Mono-trait (HTMT) ratio was the indicator used to measure the discriminant validity, which has a recommended range of below 0.9 (Taherdoost, 2016). The results for the HTMT ratio across all measures in Table 5 are significantly lower than 0.9, confirming that there is less correlation between items within the same construct than between items across constructs. As a result, the discriminant validity is still valid. Also evaluated using the Fornell-Larcker criterion was the discriminant validity depicted in Table 6. Except for the balance work-life measure, the correlations among the other constructs were more than the diagonal in italics representing the root of squares for each construct’s AVE.

**Table 5:** Fornell–Larcker Criterion (FLC) ratio of measures.

Variables	WE	WB	MC	IE	IA	AVE	FLC
WE	0	0.1832	0.0932	0.1622	0.2733	0.6101	‘Satisfied’
WB	0	0	0.6022	0.1954	0.2881	0.5140	‘Not Satisfied’
MC	0	0	0	0.2021	0.279	0.6974	‘Satisfied’
IE	0	0	0	0	0.3272	0.6570	‘Satisfied’
IA	0	0	0	0	0	0.5852	‘Satisfied’

Source: Authors’ calculation.

**Table 6:** Estimation of maximum likelihood of measures.

Fitness index	Estimation value	Model fit
SRMR	0.076	Excellent Fit
NFI	0.867	Adequate Fit
GFI	0.843	Adequate Fit
TLI	0.893	Adequate Fit
IFI	0.927	Excellent Fit
CFI	0.925	Excellent Fit

Source: Authors’ calculation.

### 4.3 Structural model

Initially, a homogeneity test was performed to ensure the statistical assumptions between predictors or independent variables and outcomes or dependent variables, which is vital to examine the validity and accuracy of the data. The homogeneity of variance for the data across the different groups has been tested and established, demonstrating that the equal variance assumptions are validated for the predictors or independent variables— AI managerial capability, AI individual expertise, and AI infrastructure agility—as well as the outcomes or dependent variables— women empowerment and work-life balance. Classically, this is measured using Levene’s Test, and *p*-values are found to be insignificant (> 0.05) recommends that the variances are insignificant across the different groups, thus ensuring homogeneity.

All five variables and the associated construct were endangered to confirmatory factor analysis (CFA) to examine the model’s hypotheses. The CFA findings showed that there was little difference between the assumed and observed models. Table 7 shows that the value of the Standardized root mean square residual (SRMR) was 0.076 (Ringle et al., 2020), and according to Hu and Bentler (1998), a value below 0.08 is generally considered to be an indicator of an excellent model fit. The anticipated hypothetical model of the research produced a satisfactory fit, as shown by the Normed Fit Index (NFI) value of 0.867 (Bentler, 1990). A value of 0.843 and 0.898 for the Goodness of Fit Index (GFI) (Wang et al., 2020) and Tucker-Lewis Index (TLI) (Shi et al., 2019) showed that the study’s proposed theoretical model produced a good fit. Also, the values of 0.927 and 0.925 for the Increment Fit Index (IFI) (Widaman and Thompson, 2003) and Comparative Fit Index (Bentler, 1990) showed that the proposed theoretical structure for investigation produced a very good fit.

**Table 7:** Analysis of measures for regression (H_1_, H_2_, and H_3_).

Dependent variables	Independent variables	*R*	*R* ^2^	Adj *R*^2^	β	*T*	*F*	Sig.
WE_WB	(constant)	0.673^a^	0.453	0.443		2.671	48.512	0.000^b^
	MC				0.320	5.060		0.000
	IE				0.043	0.689		0.487
	IA				0.319	5.170		0.000

Source: Authors’ calculation.

The outcomes of the immediate connection or path analysis of the latent factors are displayed in Table 8. Hypothesis 1 was confirmed, which suggests that the balance of work-life and empowerment of females are greatly impacted by managerial AI capabilities. Hypothesis 2 was not accepted, which confirms that AI individual expertise does not influence the balance of work-life and empowerment of females. The significance of the relationship between the agile AI infrastructure, empowerment, and work-life balance of women was confirmed by accepting Hypothesis 4 as well.

**Table 8:** Measures for regression (H_4_).

Dependent variables	Independent variables	*R*	*R* ^2^	Adj *R*^2^	β	*T*	*F*	Sig.
WE	(constant)	0.460^a^	0.211	0.207		2.790	47.645	0.006
	WB				0.579	6.090		0.000

Source: Authors’ calculation.

Empowerment and Work-Life Balance Women, the dependent variable in this analysis model, has multiple regression coefficients (R) with a fair level of predictability of 0.673. The coefficient of determination, or R2, is 0.453, as shown in Table 8. The model’s ability to satisfactory completion of 45.3% of the independent variables was made clear by this.

The F-ratio now supports the data fit of the aggregate regression system. The fact that the independent variables (MC, IE, and IA) statistically forecast the variables of the dependent (WE_WB) is an indication that the data is well set in the model fit of regression. *F* (3, 180) = 48.512, the sum of squares 53.737, which depicts the difference ascribed to the fault, and p levels suggest the independent variables (MC, IE, and IA) do so.

Empowerment and balance of work-life of females are concerning the common coefficients of regression for MC (*β* = 0.320, *p* = 0.000), IE (*β* = 0.043, *p* = 0.487), and IA (*β* = 0.317, p = 0.000). According to the correlation as well as significance findings, these characteristics are favorably and substantially connected with the empowerment and work-life balance of women.

Table 9 displays the findings that directly correlate or route analysis between the constructs of Work-Life Balance (WL) and Women Empowerment (WE). The significance of the connection between the balance of work-life and the empowerment of females was confirmed by hypothesis 4, which was also accepted. Below 0.005 is the significance level (sig.) As a result, shows that the balance of work-life and empowerment of women are significantly related.

**Table 9:** Moderation analysis results (H5 to H8).

Hypothesis (H5, H6)			Age (in Years) (H5)					Education level (H6)			
			Path coefficient				Path coefficient difference	Path coefficient			Path coefficient difference
			21 to 30	31 to 40	41 to 50	> 50		UG	PG	Professionals	
H1	MC	WE_WB	0.223**	0.341**	0.267**	0.114	0.093	0.217**	0.225**	0.274**	0.173
H2	IE	WE_WB	0.331**	0.224**	0.047	0.244**	0.141	0.287**	0.272**	0.312**	0.182
H3	IA	WE_WB	0.089	0.211**	0.028	0.236**	0.276**	0.117	0.314**	0.174	0.291**
H4	WB	WE	0.276**	0.145	0.373**	0.287**	0.098	0.092	0.045	0.192	0.027

Hypothesis (H7, H8)			Work experience (in Years) (H7)					Marital status (H8)		
			Path coefficient				Path coefficient difference	Path coefficient		Path coefficient difference
			< 1	1 to 5	5 to 10	> 10		Married	Unmarried	
H1	MC	WE_WB	0.256**	0.214**	0.334**	0.114	0.143	0.253**	0.145	0.142
H2	IE	WE_WB	0.154	0.116	0.112	0.102	0.023	0.294**	0.233**	0.046
H3	IA	WE_WB	0.247**	0.098	0.034	0.032	0.178	0.284**	0.283**	0.113
H4	WB	WE	0.365**	0.265**	0.224**	0.221**	0.125	0.371**	0.311**	0.145

Source: Authors’ calculation. **p-value less than 0.05.

### 4.4 Moderation analysis for age, education level, experience, and marital status

Multigroup analysis (MGA) was performed to examine the moderating relationship of age (H5), education level (H6), experience (H7), marital status (H8) with AI managerial capability, AI individual expertise, and AI infrastructure agility with women’s empowerment and work-life balance (H1 to H4). Table 9 represents the mediating analysis between AI managerial capability, AI individual expertise, and AI infrastructure agility with women’s empowerment and work-life balance vary significantly across different age-wise classifications, education levels of the respondents, work experience in years, and marital status of the respondents. For age groups, the path coefficients reveal that the relationship between AI managerial capability and women empowerment and work-life balance is significant and strongest for the 31 to 40 years age group of the respondents, with a path coefficient of 0.341, while it weakens and insignificant the relationship for the above the 50-age group (0.114). The finding emphasizes that older people who are about to retire from their work would not show in implementing AI. The age group of 31–40 is expected to perform well in both work and family. Therefore, the impact of AI is strongly impactful for them as per expected. The impact of AI individual expertise on women’s empowerment and work-life balance is most noticeable in the 21 to 30 years age group (0.331) but moderates significantly in the 41 to 50 years age group (0.047). This result is in line with the findings of Melweth et al. (2024). They found that experience and gender were highly correlated with burnout and technology confidence among secondary school teachers. Regarding education level, professionals show a stronger relationship between MC and WE_WB (0.274) compared to those with undergraduate degrees (0.217). The influence of work experience on these relationships also varies, with the strongest impact of AI managerial capability on women empowerment and work-life balance observed in respondents with 5 to 10 years of work experience (0.334). Finally, the marital status of the respondents moderates these relationships, predominantly for AI individual expertise influence on women empowerment and work-life balance, which is stronger and more significant among married individuals (0.294) than unmarried individuals (0.233). Overall, these results emphasize the significance of considering demographic factors when analyzing women’s empowerment and work-life balance as outcomes.

## 5 Conclusion, discussion, limitation, and future implications

### 5.1 Conclusion and discussion

The significance of AI grows more obvious given the outline of the numerical transition becomes more distinct. The aptitude to associate work and life is crucial for working women. The unpredictability of technological environments makes it difficult for women working in this field to combine their multiple and triple responsibilities. Because of problems in juggling work and life, many female professionals quit their jobs. To understand these issues, this research observes the implications of AI capabilities on the balance of work-life and empowerment of female faculties concerning educational institution efficiency. With the support of the women faculties in Saudi Arabia Universities, the data were gathered. In conclusion, the outcome shows that AI managerial capability and AI infrastructure agility have beneficial impacts on empowerment for women and the balance of work-life of female faculties.

In recent years, huge data have become more accessible, and advanced tools and infrastructure have arisen, making AI a major technology concern for organizations. To improve process-level efficiency within an organization. The benefits of AI-enabled transformation programs for businesses will be a focus of this investigation. This study uses recent research on AI in the organizational setting as well as previous technological capacity literature to build the idea of AI ability. This research identifies various essential types of artificial intelligence capabilities and then classifies them into three categories: AI managerial capability, AI individual expertise, and AI infrastructure agility. Our research builds on previous past and current research on AI in the educational institution context.

This study underscores the significant impact that AI capabilities have on the work-life balance and empowerment of female faculty members in Saudi Arabian educational institutions. Specifically, the findings demonstrate that both AI managerial capability and AI infrastructure agility positively influence these outcomes, suggesting that AI can be a powerful tool for supporting women in their professional and personal lives. The results show that AI managerial capability plays a crucial role in empowering female faculty members by enhancing their ability to manage tasks more efficiently. This aligns with prior research indicating that AI-driven management tools can improve decision-making processes, reduce administrative burdens, and provide more flexibility in managing work responsibilities (Choi, 2021; Heath, 2021; Melweth et al., 2024; Murugesan et al., 2023). By streamlining workflow and enabling more strategic allocation of time and resources, AI managerial tools contribute to a more balanced integration of work and life for women in academia.

In addition, the study also reveals that AI infrastructure agility, which refers to an institution’s ability to adapt AI technologies to changing needs, has a significant impact on the work-life balance of female faculty members. This finding is consistent with existing literature that highlights the importance of adaptable and responsive technological environments in supporting employee well-being (Dubey et al., 2021; Giraud et al., 2023). An agile AI infrastructure allows for the customization of work processes to better align with individual needs, thereby enhancing both personal and professional empowerment.

Despite the benefits of AI, the study also highlights ongoing challenges in achieving work-life balance for women in academia, particularly in technologically advanced environments. The unpredictable nature of AI and other technological advancements can add complexity to already demanding roles, exacerbating the difficulty of balancing professional duties with personal responsibilities. This challenge is well-documented in the literature, where the stress associated with rapid technological change is often cited as a barrier to work-life balance, especially for women who manage multiple roles (Alkinani, 2021; Ata et al., 2022; Mahmood et al., 2023).

The result also suggests that institutions must recognize the value of balancing work and life for their employees and the empowerment of female workers. Therefore, firms may make the proper adjustments to authorize their female aptitude and help to achieve an improved balance of work-life (Muafa and Al-Obadi, 2024). Consequently, the research advises that the Saudi Arabian government as well as its policymakers take this into account and start training programs to inspire and support families in setting objectives for their young women. Additionally, executives in charge of institutions have to look at the advantages of combining different AI technologies, it may improve female empowerment and a healthy work-life balance (Begum et al., 2024; Iyer et al., 2024).

### 5.2 Future implications and limitations

However, the challenges of gender prejudice, stereotypes, and the struggle to achieve work-life balance persist. These issues continue to limit women’s professional development and engagement, indicating that while AI offers opportunities for empowerment, there are still significant barriers that need to be addressed.

Three recommendations are designed to address the challenges identified in this research, with a focus on promoting gender equality, enhancing work-life balance, and supporting the professional development of women.

1. Support work-life balance initiatives: The government and institutions should encourage flexible work arrangements and remote work opportunities for female faculty members in educational institutions. These arrangements can help women manage their professional and personal responsibilities more effectively. Indeed, by introducing AI-driven tools and resources designed to assist women in balancing work and personal life, these could include AI-powered time management apps, mental health support, and platforms for virtual collaboration.
2. Tailor interventions to demographic factors: it is important to design targeted interventions that consider the specific needs of women based on their age, education level, experience, and marital status. For instance, younger women or those with less experience may benefit from mentorship programs, while experienced women might need leadership development opportunities. Institutions may adapt their policies to better support diverse groups of women by conducting regular assessments of the impact of AI on work-life balance and women’s empowerment, with attention to demographic differences.
3. Enhance AI infrastructure in educational institutions: Invest in AI infrastructure that supports the professional development of female faculty members perform AI tools for personalized learning, research facilitation, and administrative efficiency and ensure that AI systems within educational institutions are designed and implemented with gender-sensitive approaches.

Finally, the research has certain limitations, which offer guidance for future investigation. First off, since the information was gathered in the particular geographic region of one nation, upcoming research should contain more nations with diverse economic conditions to better corroborate the findings. Besides this, respondents were reluctant to give their own opinions through answering the questionnaire due to job insecurity that management might know their given answers. Samples were taken from educational sectors in Saudi Arabia. Further research can be done by focusing on various job sectors and also by increasing the sample size. Finally, a separate study background with distinct study questionnaires needs to be created to perform throughout a variety of service sectors, such as IT industries, hospitals, and hotels for conventions. These insights suggest that AI when harnessed effectively, can contribute significantly to improving the efficiency and effectiveness of educational institutions by empowering women and supporting their well-being.

## Data Availability

The original contributions presented in the study are included in the article/supplementary material, further inquiries can be directed to the corresponding author.
